# Cure of Arterio Venous Aneurism by Extirpation

**Published:** 1890-07-12

**Authors:** 


### CURE OF ARTERIO VENOUS ANEURISM BY EXTIRPATION.

Dr. Menocal, in *El Siglo Medico*, gives an interesting account of the extirpation of a large arterio venous aneurism. A man had received a gun-shot wound in the arm, the bullet making a complete tunnel, and coming out about six cms. above the outer condyle of the humerus. There was profuse hæmorrhage, which was arrested by a tourniquet, a huge hæmatoma occupying the lower half of the arm anteriorly. Carbolised compresses were applied, and the wound healed rapidly, the hæmatoma disappearing in twelve or fourteen days. But from the first pulsation had been felt in the region of the wound, and now that the swelling had subsided the suspected aneurism was distinctly visible. When the case came under Dr. Menocal's observation the aneurism was the size of a large apple, and pulsated strongly. The vessels were much dilated, and pressure on the brachial artery did not affect the objective symptoms, while compression of the arm above increased the size of the tumour. The veins, too, were enormously swollen.

Having driven the blood out of the limb by Esmarck's apparatus, Dr. Menocal made a longitudinal incision in the skin 16 cms. long, following the course of the brachial artery from the middle of the arm to the junction of the upper and middle thirds of the forearm. He then tied the median and basilic veins, and the brachial artery at each extremity of the incision. He next extirpated the sac, together with three vessels communicating therewith, tying them where divided, and closed the wound with silver sutures. Healing took place by first intention, and the sutures were removed on the seventh day, when the cure was complete.

